# Challenges in health risk assessment of multiple chemical exposures in epidemiological studies

**DOI:** 10.1265/ehpm.23-00312

**Published:** 2024-02-08

**Authors:** Chiharu Tohyama, Yasushi Honda

**Affiliations:** 1 The University of Tokyo, 7-3-1 Hongo, Tokyo 113-8654, Japan; 2 Health, Environment, Science, and Technology International Consulting, 5-9-7 Toyotama-kita, Nerima-ku, Tokyo 172-0012, Japan; 3 The National Institute for Environmental Studies, 16-2 Onogawa, Tsukuba 305-8506, Japan; 4 Univeristy of Tsukuba, 1-1-1 Tennou-dai, Tsukuba 305-8577, Japan

Developed countries have entered a population decline phase, with the total fertility rate lower than that required to preserve the species sustainably [1]. The trend is apparent in the decades since 1960 [2] and coincides with worsening global chemical pollution, an indicator of the Anthropocene, alongside climate change and other factors [3]. In addition to socioeconomic and cultural factors, male reproductive abnormalities associated with global chemical pollution have been postulated as a contributing influence [4].

The concept that exposure to high concentrations (or high doses) of xenobiotic environmental contaminants can cause organic and functional abnormalities in the reproductive organs has been established by epidemiological and clinical findings in industrial settings and pollution incidents, as well as by reproductive developmental toxicity studies using laboratory animals. However, whether and how exposure to low concentrations (or low doses) of xenobiotic chemicals causes adverse effects on male reproductive function through endocrine disruption remains an issue that requires scientific verification.

In 1992, Carlsen et al. [5] reported that sperm concentrations had halved in men in the 50 years between 1938 and 1991. This and other studies indicated that endocrine-disrupting chemicals are suspected to be responsible for the decline in semen quality [4, 6]. The declining trend of semen quality has been confirmed in systematic reviews addressing downward trends of sperm concentration and total sperm count in North American, European, and Australian populations [7] and Latin American, Asian, and African populations [8] in the period 1981–2013. A recent study has countered, however, that generalizing the decline in spermatogenic potential in specific populations should be approached cautiously due to methodological issues [9]. Further research will be necessary to reach a consensus.

Prospective child cohort studies could be a highly effective methodology for this purpose. Here, we first review a series of studies based on a child cohort to show how effective this series is and then discuss how we can best utilize the academic results for policymaking. A prospective cohort study that has been conducted by a research group at Harvard School of Public Health (Russian Children’s Study, hereafter) since 2003 has found a significant relationship between exposure to low concentrations (or low doses) of environmental contaminants and health outcomes (mainly abnormal reproductive development in boys) and partly contributes to real-world environmental health policy. Indeed, the EFSA’s Panel on CONTAM proposed dioxin tolerable intakes that are approximately ten times lower than prior guidelines, based on the original paper from the Harvard research group [10, 11]. The details are discussed below, but the proposal was said to be based on exposure to a single chemical with adjustment for possible confounding effects by coexisting contaminants.

However, in 2022 and 2023, Burns and others in the same study group reported that phthalates were associated with delayed onset of puberty in the Russian Children’s Study [12, 13]. This group also published a paper showing that exposure to lead, organochlorine pesticides, and other chemicals is involved in delayed puberty onset (see the references cited below). Thus, in addition to dioxins, simultaneous exposure to several chemicals involved in delayed onset of puberty had occurred. While determining the effects of each chemical is important, additional valuable insight would be gained if the combined exposure to all known causative agents were considered. Details are provided below.

The Russian Children’s Study is a longitudinal cohort study of preadolescent and postadolescent boys and their mothers, residents of Chapaevsk, a regional city in Russia, 950 km southeast of Moscow. In this city, large-scale production of chemical warfare agents (before 1949) and industrial and agricultural chemicals containing chlorine (hexachlorocyclohexane (from 1967 to 1987), hexachlorobenzene, PCP, methylchloroform, vinyl chloride, and others) was carried out [14]. These manufacturing processes and waste incineration resulted in extensive local contamination with PCDD/Fs, lead, and other chemicals. A total of 516 boys aged 8–9 years (as of 2003–2005) living in the city, representing approximately 90% of the total number, were surveyed to determine the effects of environmental contaminants (dioxins (TCDD, PCDD/Fs and coplanar PCBs), organochlorine pesticides, lead, and phthalates) on their growth and sexual maturation at age 18–19 (as of 2013–2015). At the enrollment and subsequent follow-up examinations, boys and their mothers were interviewed, physically measured, and had blood samples taken, with their consent. In addition, semen was collected from those boys aged 18–19 with their consent. The main findings of reports from the Russian Children’s Study on the relationship between environmental contaminants and the onset of puberty are as follows.

1. Relationship between lead and onset of puberty. In boys aged 8–9 years, the median blood lead concentration was 3 µg/dL, with significant inverse association with both height and weight. It was concluded that boys aged 8–9 years with blood lead levels of 5·0 µg/dL or higher had a later onset of puberty (i.e., testicular volume >3 ml, genital stage 2 or higher, or pubic hair stage 2 or higher) than boys with lower lead exposure [15, 16]. Preliminary findings in 125 boys also suggested that dioxin exposure is unlikely to affect the finding of delayed onset of puberty due to lead, as there is no significant correlation between serum dioxin levels and blood lead levels [15].

2. Dioxin exposure levels in mother-child pairs. Total serum dioxin (PCDDs, PCDFs, coplanar PCBs, and other PCBs) concentrations (median) in 8- to 9-year-old boys were 21·1 pg TEQ/g lipid [17]. Among 446 mothers (23–52 years) in the study, total PCB and total TEQ concentrations (median) were 260 ng/g lipid and 25·0 pg TEQ/g lipid, respectively [18]. These concentrations were significantly correlated with maternal age, distance to the chemical plant, length of time working on gardening, and local beef consumption [17], and it was concluded that children tended to have higher levels of serum dioxins [18]. An association was found between maternal serum total PCB levels and earlier puberty in boys, but no consistent trend was found with total TEQ levels [19].

3. Relationships of dioxin exposure levels with semen quality [10]. The relationship of dioxin exposure levels during preadolescence (8–9 years) with the semen quality (sperm concentration, total sperm count, and total motile sperm count) of adolescent boys (18–19 years) was examined. The median and range of serum TCDD concentrations and PCDD TEQs were 2·9 (0·4–12·1) pg/g lipid and 8·7 (1·0–36·0) pg/g lipid, respectively. With higher values of these parameters, a trend towards significantly lower semen quality was observed. However, no significant associations were observed between other parameters (serum PCDFs, PCBs concentration, and total TEQ conversion) and semen quality.

Using this observation, the European Food Safety Agency’s (EFSA’s) Expert Panel on contaminants in the food chain (CONTAM) derived a tolerable weekly intake (TWI) of 2·0 pg TEQ/kg per week [11], with a note saying that the effects of TCDD and PCDD on delayed onset of puberty were observed when lead and organochlorine pestiside (HCB, βHCH, p-p′ DDE) exposure was adjusted. However, the original paper [10] does not state whether and how this adjustment was made.

4. Relationship between organochlorine pesticides and puberty onset and sexual maturation. The authors analyzed the relationship of serum organochlorine pestiside (HCB, βHCH, and p-p′ DDE) concentrations obtained in preadolescence (8–9 years) with the start of puberty [20] or sexual maturation [21] in 350 boys (16–17 years) 8 years after the start of the cohort. Serum HCB concentrations (median) were 158 ng/g lipid, more than ten times higher than the median value for children in the USA. Higher HCB concentrations tended to delay the onset of puberty (testicular volume >3 mL and >pubic hair stage P2). In contrast, no significant relationship was found between serum βHCH and p-p′ DDE concentrations and the timing of the onset of puberty., Serum HCB and βHCH concentrations were correlated with delayed sexual maturation (testicular stage 5, pubic hair stage 5, testicular volume >20 mL). It should be noted that HCB is a known AhR agonist. There is no mention in this paper of the effects of dioxins, PCBs, lead concentrations, or other chemicals.

5. Relationship between phthalates and the onset of puberty. In 2022 and 2023, Burns and others published papers [12, 13] in which they analyzed the relationship of urinary concentrations of metabolites of several antiandrogenic phthalates (BBzP, DnBP, DiBP, DEHP, and DiNP) at age 8–9 years old with male genital development after ten years (18–19 years old). In the 2022 paper [12], the authors found that elevated urinary concentrations of phthalates’ metabolites (individual and total concentrations) were associated with an 8–14 months delay in reaching pubic hair stage P2, and several of the same metabolites were associated with the delay of testicular developmental stage G2 and testicular volume > 3 mL by 5·4–7·5 months. The 2023 paper [13] examined the effect of phthalates on year-to-year testicular development. It inferred that one metabolite, MBzP, suppressed testicular development by disturbing androgen-dependent pathways. It also notes weak correlations between serum dioxins TEQ, total PCBs, organochlorine pesticides (HCB, βHCH, p-p′ DDE), and blood lead, respectively.

It was reported that, when analyzing the effects of certain chemicals covered in this cohort study, other coexisting chemicals were statistically adjusted to analyze whether and to what extent the chemical in question had an effect. It should be noted that the EFSA’s risk assessment for dioxins [11] set tolerances without adjustment for phthalates, for which the results were only published 4–5 years later. In addition, another coexisting chemical (e.g., paraben) is planned for future analysis by the same study group [13]. If so, the overestimation of the tolerable intake value could be greater [11].

In summary, the 10-year Russian Children’s Study of preadolescent boys adds to previous literature showing that exposure to lead, TCDD, PCDD TEQ, HCB, βHCH, and phthalates (examined so far) in preadolescence is responsible for delaying the onset of puberty and suppressing male genital development through multiple mechanisms.

It is common practice for epidemiological studies to assess the risk of specific chemicals while controlling for other coexisting chemicals, but often without reporting the risks associated with chemicals outside the focus of the study. With the perspective of protecting health from environmental chemicals, when there are multiple possible causative agents, it is necessary to use models that include as explanatory variables all types of chemicals that are assumed to be involved in multiple health outcomes. However, existing linear models usually have a limited number of variables, and there is a problem of multicollinearity, where high correlations among explanatory variables make the results unstable. To overcome such problems, a workshop “Statistical Approaches for Assessing Health Effects of Environmental Chemical Mixtures in Epidemiology Studies” was held by the National Institute of Environmental Health Sciences (NIEHS) in 2015 [22]. Here, many new methods that can handle multiple exposures and multicollinearity were proposed, but none of them outperformed the others. Following this workshop, NIEHS initiated the PRIME (Powering Research through Innovative Methods for Mixtures in Epidemiology) program [23]. This initiative included six projects and resulted in 37 new methods and software for these methods. Although the development of these methods is ongoing, they should provide a good starting point to evaluate the impact of multiple chemical species in a certain study population.

Although the Russian Children’s Study findings are invaluable, they are limited to a single cohort. To see how effects may differ in different populations, it is advisable to conduct meta-analyses on several different target populations. This would allow risk assessment and regulatory prioritization according to the exposure situation in each cohort. Determining the consistency of risk assessments of the same chemical and health outcomes reported from multiple studies that utilize different analytical methods is also challenging. Therefore, organizing consortia and performing meta-analyses intended to measure chemicals using the same techniques and assess their association with identically defined outcomes in multiple populations can be highly effective.

In recent years, birth cohort research projects on health and the environment have been conducted in many countries. In Japan, a national project called the Japan Environment and Ecological Children’s Study [24], involving 100,000 mother-child pairs, was initiated in 2011 to examine the central hypothesis that environmental factors, including chemical exposure from fetus to childhood, affect pregnancy and reproduction, congenital malformations, neuro- and psycho-development, immunity and allergy, metabolism, the endocrine system, and other endpoints. Many original papers have already been published, but most are on the relationship between individual environmental factors and health outcomes, and integrative analysis of multiple exposure factors together has yet to be performed. In Asian countries, including Japan, the Birth Cohort Consortium of Asia has been formed to exchange information and conduct collaborative research [25]. For example, a pooled analysis of both Taiwanese and Korean cohorts found an inverse association between maternal and umbilical cord blood mercury levels and birth weight [26]. We would expect that such collaboration will deepen and spread throughout the world.

Based on the discussion above, we suggest that research consortia can ideally contribute to the evaluation of the risks of multiple chemicals in three ways: (1) They can establish uniform exposure and outcome definitions; (2) they can provide useful methods for assessing multiple chemical exposures, as the PRIME project [23] has done; and (3) they can perform meta-analyses of results across cohorts to provide information on the consistency of results.
